# A particular focus on the prevalence of α-thalassemia and β-thalassemia among pregnant women in Changsha County, Hunan Province

**DOI:** 10.3389/fgene.2024.1422462

**Published:** 2024-11-07

**Authors:** Yu Xia, Cailian Huang, Mudan Yang, Meng Zhang, Yang Lu

**Affiliations:** Department of obstetrics and gynecology, Changsha County maternal and child health hospital, Changsha, Hunan, China

**Keywords:** pregnant women, next-generation sequencing, thalassemia, hematological parameters, Changsha County

## Abstract

**Background:**

Thalassemia is a inherited monogenic blood disorder and more prevalent in southern China. In this study, Our aim was to elucidate the molecular spectrum and phenotypic features of thalassemia in pregnant women in Changsha County.

**Methods:**

Next-generation sequencing (NGS) was conducted for 38,810 pregnant women to diagnose thalassemia in Changsha County. Further analysis of hematological parameters was conducted on subjects who had not previously undergone thalassemia screening in other hospitals.

**Results:**

In this study, 2,208 (5.69%) pregnant women were diagnosed as carriers of thalassemia using NGS analysis. Among 1,594 cases of α-thalassemia, 23 genotypes were identified, and among 578 cases of β-thalassemia, 22 genotypes were detected. Additionally, 18 genotypes were detected among 36 cases of composite α- and β-thalassemia. Among all carriers of thalassemia, 8 rare α-mutations and 11 rare β-mutations were found in the study population. Notably, pregnant women diagnosed as carriers of thalassemia tended to have lower hemoglobin levels. Furthermore, multivariable logistic regression analysis indicates that the values of MCV and MCH have the greatest impact on genetic diagnosis.

**Conclusion:**

Our study has provided detailed genotypes and hematological parameters of thalassemia in pregnant women in Changsha county and reveal that certain abnormal blood parameters have a remarkably impact on genetic diagnosis results. Furthermore, our data suggest that combining hemoglobin electrophoresis and NGS provides a powerful tool for prenatal diagnosis, which will increase the accuracy of clinical diagnosis of thalassemia.

## 1 Introduction

Thalassemia is a group of inherited monogenetic blood disorders caused by a defect in the globin gene, which leading to microcytic anemia, chronic hemolysis, iron loading, and even transfusion dependence in particularly severe forms (Taher et al., 2018; Harteveld et al., 2022; Sabath, 2023). Among the genotype of defective gene, thalassemia can be divided into α-, β-, γ-, δ-, δβ-, and εγδβ-thalassemias (Higgs et al., 2012). Among them, α-thalassemia (α-thal) and β-thalassemia (β-thal) are the two main forms of thalassemia, which compose a tetramer of hemoglobin (Hb) to deliver oxygen to the tissues (Ahmed et al., 2020). Clinically, thalassemia can be divided into mild (thalassemia gene carriers), intermediate and severe thalassemia based on anemia status, depending on the type and amount of hemoglobin synthesized (Shang and Xu, 2017). The severity of the anemia is closely linked to the underlying genotype, although various molecular and environmental factors may also play a role (Taher et al., 2018; Taher et al., 2021).

Thalassemia is considered one of the top five major birth defects (Dong et al., 2019), and severe thalassemia pose a significant threat to the lives and health of newborns per year (Ip and So, 2013). To date, there is no effective treatment for thalassemia except for gene therapy hematopoietic stem cell transplantation (Philippidis, 2023; Snowden et al., 2022), and early diagnosis of thalassemia and carrier screening are crucial measures to timely prevention and treatment of thalassemia (Goonasekera et al., 2018). To prevent ineffective and possibly detrimental interventions, as well as to provide genetic counselling in case of planned pregnancies, it is necessary to implement the genetic testing in the pregnant women during the first and second trimesters (Piel and Weatherall, 2014; Rund and Rachmilewitz, 2005). The genetic information is vital for the effective management of individuals with thalassemia.

The prevalence of thalassemia had obvious regional differences. This region-specific stretches from the Mediterranean to Southeast Asia and south through Sub-Saharan Africa (Weatherall and Clegg, 2001; Farmakis et al., 2020). In China, the incidence of thalassemia also varies greatly among different provinces, and the population in different regions has their own spectrums of thalassemia (Tang et al., 2015). While several studies have been reported thalassemia is highly prevalent in some provinces of China, such as Hainan, Guangdong, Guangxi and Hunan Province (Yao et al., 2014; Zhao et al., 2018; Xiong et al., 2010; Zhang et al., 2019). Unfortunately, data on the population prevalence of hemoglobinopathies in pregnant women remain limited. Most reports have been conducted at the provincial level, which depict a low proportion of people undergoing screening (Zhang et al., 2019; Luo et al., 2022). Changsha county, with a permanent population of 1.4 million, and our screening covers almost all pregnant women. Furthermore, most study only focus on the molecular spectrum of thalassemia, without delving into the correlation between genetic and phenotypic features in carriers of α- and β-globin genetic variants. Therefore, further analysis of the genetic nature and blood cell characteristics of these disorders to enhance the clinical management of genetic disorders of haemoglobin is required (Pan et al., 2007; Daidone et al., 2023).

Here, we analyzed the genotypes of α- and β-thalassemia in pregnant women who underwent thalassemia screening (routine blood tests and hemoglobin electrophoresis) and genetic detection in Changsha county. We aimed to determine the true prevalence, gene distribution, and phenotypic features of thalassemia in pregnant women in this area. The findings from our research may provide a theoretical bases for genetic counseling and clinical measures for the prevention and management of thalassemia.

## 2 Materials and methods

### 2.1 Study subjects

This study was approved by the Ethics Committee of Changsha County Maternal and Child Health Hospital (No. XFYIRB-2024001). From September 2017 to March 2023, the genetic diagnosis results of 38,810 pregnant women were retrospectively collected in Changsha County Maternal and Child HealthCare Hospital, Hunan, China. The participants, age from 18 to 56 years, provided written informed consent before peripheral blood samples were collected.

### 2.2 Hemoglobin testing

A measure of 2 mL of peripheral venous blood samples was collected in ethylene diamine tetraacetic acid K2 (EDTA-K2) anticoagulated tubes for screening and genetic diagnosis of thalassemia. Red cell indices were detected using a Sysmex XN-1000 automatic blood cell analyzer (Kobe, Japan) according to the standard operating procedure. Participants with reduced mean corpuscular volume (MCV) < 80 fL and/or reduced mean corpuscular hemoglobin (MCH) values <27 pg were considered suspected thalassemia carriers. Approximately 16,223 (41.8%) subjects who had undergone thalassemia screening at other hospitals did not receive the blood routine test and were only subjected to molecular diagnosis.

### 2.3 Genetic analyses of thalassemia

According previously reported method (Luo et al., 2022). Genomic DNA was prepared from 200 μL whole blood samples using the QIAamp DNA Blood Mini Kit (Qiagen, Hilden, Germany). The genomic DNA of the subjects was isolated by the GenMag Nucleic Acid Isolation kit (Magnetic bead method) (GenMagBio, Beijing, China). The concentration and purity of gDNA were assessed by the NanoDrop-8000 spectrophotometer (Thermo Scientific, Waltham, MA, United States). A combined strategy of Gap-PCR and NGS was applied to detect thalassemia. Briefly, three α-thalassemia gene deletions (--^SEA^/, -α^4.2^/, and -α^3.7^/), and four β-thalassemia gene deletions (SEA-HPFH, Chinese ^G^γ + (^A^γδβ)^0^, Taiwanese deletion) was tested by the Gap-PCR. Other mutations in globin genes were analyzed by NGS. The target sequences of HBA1, HBA2, and HBB were firstly amplified and enriched through multiplex PCR, and then sequencing libraries were constructed by MGISEQ-2000 sequencing platform (MGI, Shenzhen, China) according to the manufacturer’s instructions. The bioinformatic analysis of identifying hemoglobin gene mutations using previously described protocol (He et al., 2017a). Briefly, after performing whole-globin gene sequencing via next-generation sequencing, the HbVar and IthaGenes databases were used to annotate the identified mutations.

### 2.4 Statistical analysis

Data and results were analyzed using R statistical software, version 4.0.2 (R Foundation for Statistical Computing). The odds ratios (ORs) and 95% confidence intervals (CIs) for assessing correlation between age, hematological parameters and incidence of thalassemia. The *p* values <0.05 were considered to indicate statistical significance.

## 3 Results

### 3.1 The genotypes of α-thalassemia

Our study enrolled a total of 38,810 subjects, among whom 2,208 cases were detected with mutations by NGS. These cases included 1,594 α-thalassemia, 578 β-thalassemia, and 36 combined α-/β-thalassemia (Table 1; Table 2; Table 3). Among the 1,594 individuals with α-thalassemia mutations, 23 α-globin genotypes were identified, in which the -α^3.7^/αα mutation was the most abundant genotype, accounting for over half of the mutations (52.76%), followed by -@-^SEA^/αα (26.91%), -α^4.2^/αα (10.35%), and α^WS^α/αα (3.95%) (Table 1). In addition, We identified 8 uncommon α-thalassemia genotypes that have a very low carrier rate in the general population in China, including -@-^THAI^/αα (3 cases), CD 61 AAG > TAG [Lys > STOP]/αα (3 cases), CD 30 -GAG [-Glu]/αα (2 cases), α^fusion^/αα (2 cases), Init CD (-T)/αα (2 cases), Hb Amsterdam-A1/αα (1 case), Hb Phnom Penh/αα (1 case), and CD 108 ACC > AAC [Thr > Asn]/αα (1 case).

**TABLE 1:** Prevalence rate and mutations spectrum of α-thalassemia alone.

Genotypes	Phenotype	Cases	Frequency (%)
-α^3.7^/αα	α^+^/α	841	2.167
-@-^SEA^/αα	α^0^/α	429	1.105
-α^4.2^/αα	α^+^/α	165	0.425
α^WS^α/αα	α^+^/α	63	0.162
α^CS^α/αα	α^+^/α	37	0.095
α^QS^α/αα	α^+^/α	20	0.052
-α^3.7^/-α^3.7^	α^+^/α^+^	9	0.023
-α^3.7^/-α^4.2^	α^+^/α^+^	4	0.010
-@-^THAI^/αα^a^	α^0^/α	3	0.008
α^31^α/αα	α+/α	3	0.008
-α^3.7^/-@-^SEA^	α+/α^0^	3	0.008
CD 61 AAG > TAG [Lys > STOP]/αα^a^	α+/α	3	0.008
CD 30 -GAG [-Glu]/αα^a^	α+/α	2	0.005
α^fusion^/αα^a^	α+/α	2	0.005
HKαα/-@-^SEA^	α+/α^0^	2	0.005
Init CD (-T)/αα^a^	α+/α	1	0.003
Hb Amsterdam-A1/αα^a^	α+/α	1	0.003
-α^4.2^/-α^4.2^	α+/α+	1	0.003
Hb Phnom Penh/αα^a^	α+/α	1	0.003
α^WS^α/-@-^SEA^	α+/α^0^	1	0.003
CD 108 ACC > AAC [Thr > Asn]/αα^a^	α+/α	1	0.003
α^QS^α/-α^3.7^	α+/α+	1	0.003
α^WS^α/-α^3.7^	α+/α+	1	0.003
Total		1,594	4.107

^a^ Indicates genotypes with rare mutations.

**TABLE 2:** Prevalence rate and mutations spectrum of β-thalassemia alone.

Genotypes	Phenotype	Cases	Frequency (%)
IVS-II-654 (C>T)/β^N^	β^+^/β^N^	205	0.528
CD 41/42 (-TTCT)/β^N^	β^0^/β^N^	115	0.296
CD 17 (A>T)/β^N^	β^0^/β^N^	74	0.191
5′UTR +43 to +40 (−AAAC)/β^N^ ^a^	β^+^/β^N^	38	0.098
CD 71/72 (A>T)/β^N^	β^0^/β^N^	27	0.070
−28 (A>G)/β^N^	β^+^/β^N^	26	0.067
β^E^/β^N^	β^+^/β^N^	22	0.057
CD 27/28 (+C)/β^N^	β^0^/β^N^	17	0.044
−50 (G>A)/β^N^ ^a^	β^+^/β^N^	13	0.033
CD 43 (G>T)/β^N^	β^0^/β^N^	9	0.023
SEA-HPFH/β^N^ ^a^	β^0^/β^N^	7	0.018
−29 (A>G)/β^N^	β^+^/β^N^	6	0.015
−72 (T>A)/β^N^ ^a^	β^+^/β^N^	6	0.015
−31 (A>C)/β^N^ ^a^	β^+^/β^N^	4	0.010
CD 43 (G>T)/β^N^	β^0^/β^N^	2	0.005
Poly A (A>G)/β^N^ ^a^	β^+^/β^N^	1	0.003
IVS II-761 (A>G)/β^N^	β^0^/β^N^	1	0.003
Chinese ^Gγ+(Aγδβ)0^/β^N^ ^a^	β^0^/β^N^	1	0.003
Taiwanese/β^N^ ^a^	β^0^/β^N^	1	0.003
IVS-II-848 (C>T)/β^N^ ^a^	β^+^/β^N^	1	0.003
CAP +8 (C>T)/β^N^ ^a^	β^+^/β^N^	1	0.003
−176(C>T)/β^N^ ^a^	β^+^/β^N^	1	0.003
Total		578	1.489

^a^ Indicates genotypes with rare mutations.

**TABLE 3:** Prevalence rate and mutations spectrum of combined α + β-thalassemia.

Genotypes	Phenotype	Cases	Frequency (%)
-α^3.7^/αα, IVS-II-654(C>T)/β^N^	α^+^/α β^+^/β^N^	5	0.013
-@-^SEA^/αα, IVS-II-654(C>T)/β^N^	α^0^/α β^+^/β^N^	5	0.013
-@-^SEA^/αα, β^E^/β^N^	α^0^/α β^+^/β^N^	3	0.008
-@-^SEA^/αα, CD 41/42 (-TTCT)/β^N^	α^0^/α β^0^/β^N^	3	0.008
-α^3.7^/αα, CD 17 (A>T)/β^N^	α^+^/α β^0^/β^N^	3	0.008
-α^3.7^/αα, CD 43 (G>T)/β^N^	α^+^/α β^0^/β^N^	2	0.005
-α^3.7^/αα, CD 41/42 (-TTCT)/β^N^	α^+^/α β^0^/β^N^	2	0.005
-α^4.2^/-α^4.2^, CD 41/42 (-TTCT)/β^N^	α^+^/α β^0^/β^N^	2	0.005
-α^4.2^/αα, IVS-II-654(C>T)/β^N^	α^+^/α β^+^/β^N^	2	0.005
-α^3.7^/αα, β^E^/β^N^	α^+^/α β^+^/β^N^	1	0.003
-α^3.7^/αα, −28 (A>G)/β^N^	α^+^/α β^+^/β^N^	1	0.003
-@-^SEA^/αα, CD 17 (A>T)/β^N^	α^0^/α β^0^/β^N^	1	0.003
-α^4.2^/αα, CD 17 (A>T)/β^N^	α^+^/α β^0^/β^N^	1	0.003
α^WS^α/-α^4.2^, CD 41/42 (-TTCT)/β^N^	α^+^/α β^0^/β^N^	1	0.003
-@-^SEA^/αα, −28 (A>G)/β^N^	α^0^/α β^+^/β^N^	1	0.003
-@-^SEA^/αα, −50 (G>A)/β^N^	α^0^/α β^+^/β^N^	1	0.003
-α^3.7^/αα, IVS-II-848(C>T)/β^N^	α^+^/α β^+^/β^N^	1	0.003
-α^4.2^/αα, IVS-II-848(C>T)/β^N^	α^+^/α β^+^/β^N^	1	0.003
Total		36	0.093

### 3.2 The genotypes of β-thalassemia

In this study, we identified 578 subjects with β-thalassemia, and 22 different genotypes were detected (Table 2). IVS-II-654 (C>T)/β^N^ is the most common genotype, accounting for 35.46% of the all β-thal carriers genotypes in this study. Other prevalent genotypes, such as CD 41/42 (-TTCT)/β^N^, CD 17 (A>T)/β^N^, 5′UTR +43 to +40 (−AAAC)/β^N^, CD 71/72 (A>T)/β^N^, −28 (A>G)/β^N^, and β^E^/β^N^, represented 19.89%, 12.80%, 6.57%, %, 4.67%, 4.49%, and 3.80% of the β-thalassemia genotypes, respectively. Additionally, a total of 74 cases with 11 rare gene types were identified. Interestingly, we observed that 38 patients had a rare genotype of 5′UTR +43 to +40 (−AAAC)/β^N^, which accounted for a high proportion of the patient population.

### 3.3 The genotypes of combined α+β-thalassemia

We identified a total of 36 cases of combined α-/β-thalassemia, with a carrier rate of 0.093% (36/38,810) in this group (Table 3). A total of 18 genotypes werre detected. The top five genotypes of αβ-thalassemia were -α^3.7^/αα combined with IVS-II-654(C>T)/β^N^ (13.88%); -@-^SEA^/αα combined with IVS-II-654(C>T)/β^N^ (13.88%), -@-^SEA^/αα together with β^E^/β^N^ (8.33%), -@-^SEA^/αα together with CD 41/42 (-TTCT)/β^N^ (8.33%), and -α^3.7^/αα together with CD 17 (A>T)/β^N^ (8.33%).

### 3.4 Allele frequencies of α-thalassemia and β-thalassemia mutations

**FIGURE 1:**
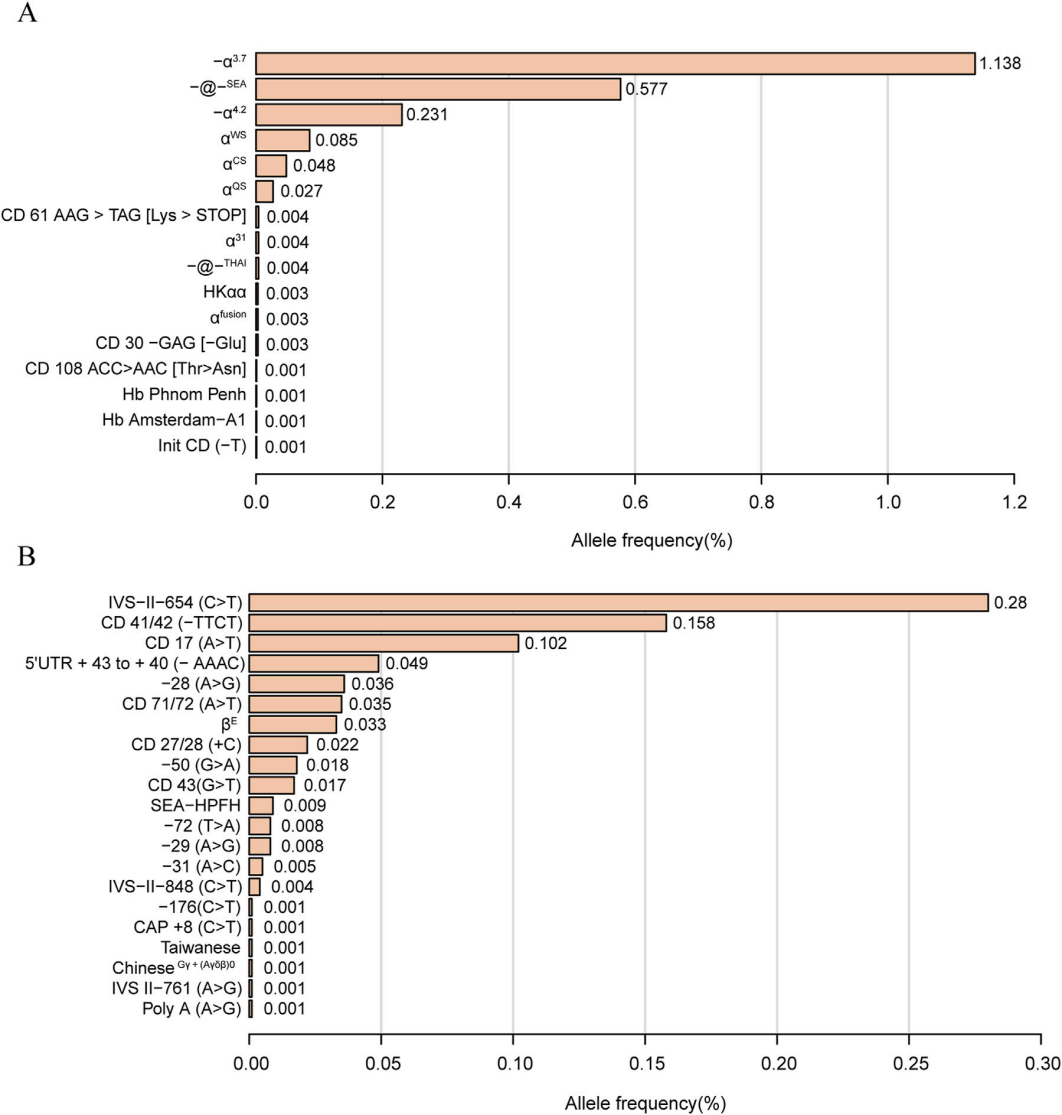
Thalassemia allele frequency distribution. α-thalassemia allele frequency distribution **(A)**. β-thalassemia allele frequency distribution **(B)**. Allele frequency = Number of alleles/total number of chromosomes investigated (38,810*2).

The allele frequencies of α-globin gene variation are shown in Figure 1. We identified 16 α-thalassemia gene mutations and 21 β-thalassemia gene mutations. Of α-globin mutant chromosomes, the most frequent types were -α^3.7^ allele, with the allele frequencies of 1.138% among all investigated chromosomes, followed by -@-^SEA^ (0.577%), -α^4.2^ (0.231%), α^WS^α (0.085%), α^CS^α (0.048%), and α^QS^α (0.027%). Of all the β-globin mutant chromosomes, the result showed the three most frequent mutations were IVS-II-654(C>T) (0.28%), CD 41/42 (-TTCT) (0.158%) and CD 17 (A>T) (0.102%).

### 3.5 Hematological parameters in positive populations

**FIGURE 2:**
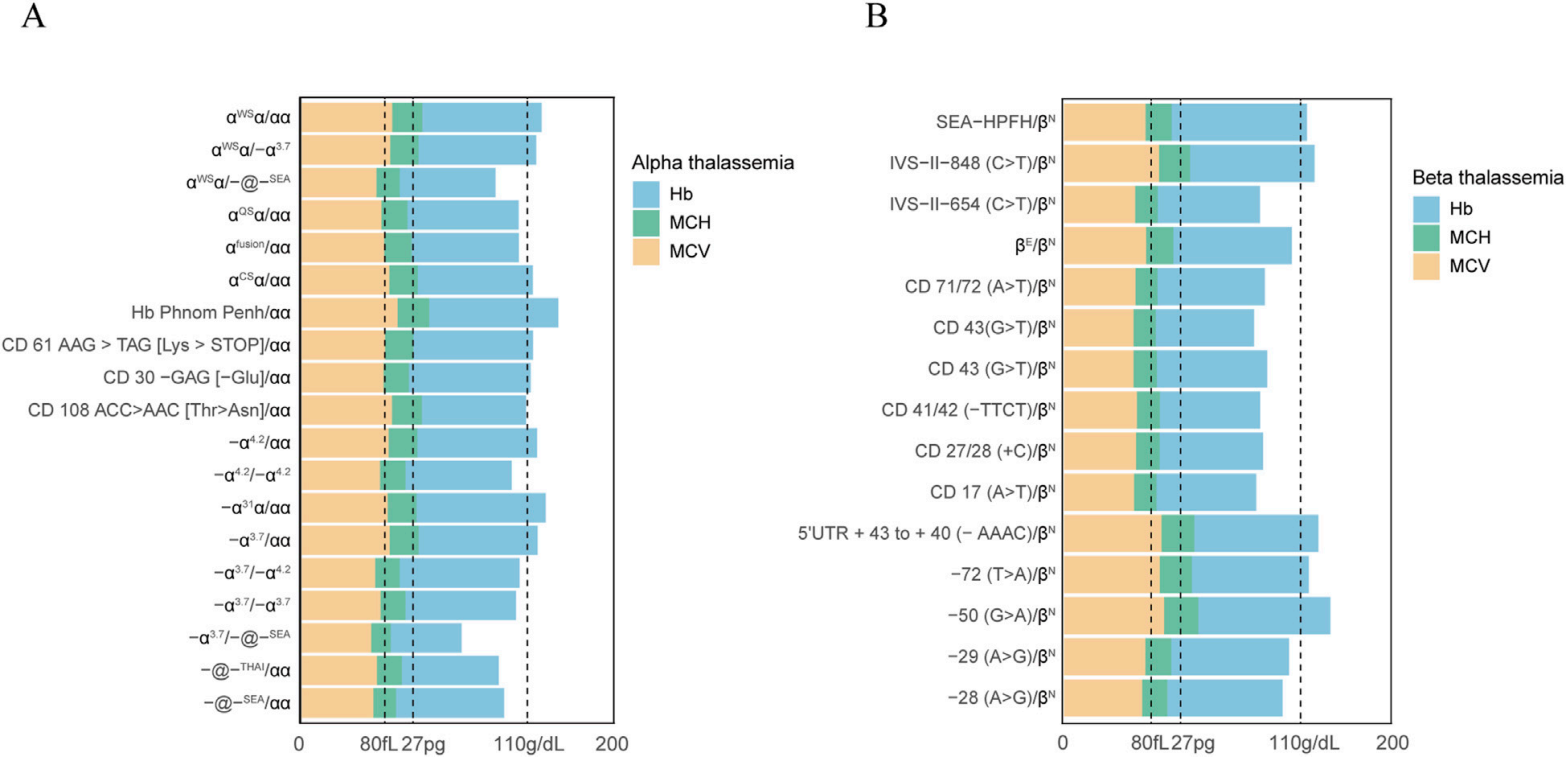
Hematological parameters observed in subjects with mutations in the α-globin or β-globin gene. Hematological parameters of all α thalassemia population **(A)**. Hematological parameters of all β thalassemia population **(B)**.

To evaluate the correlation between a given genotype and hematological parameters, haemoglobin and blood red cell parameters were analyzed in positive populations (Figure 2). Our study found that a significant number of individuals with thalassemia had a mean corpuscular volume (MCV) level below 80 fL and abnormal hemoglobin (Hb) parameters, specifically less than 110 g/dL. It is noteworthy to mention that all the positive cases in our study were women. In addition, A more marked decline in MCV values (<70 fL) and MCH (<27 pg) was observed in subjects with genotypes -α^3.7^/-@-^SEA^, α^WS^α/-@-^SEA^, IVS−II−654 (C>T)/β^N^, CD 71/72 (A>T)/β^N^, CD 43 (G>T)/β^N^, CD 43 (G>T)/β^N^, CD 41/42 (−TTCT)/β^N^, CD 27/28 (+C)/β^N^, and CD 17 (A>T)/β^N^. Interestingly, we also observed the results on MCH levels and Hb levels were highly consistent with those of MCV values. Moreover, It was observed that certain individuals with thalassemia gene mutations, such as those with genotypes α^CS^α/αα, −α^4.2^/αα, CD 61 AAG > TAG [Lys > STOP]/αα, −72 (T>A)/β^N^, had normal hematological parameters. This finding highlights the challenge of relying solely on routine hematological screening methods.

**FIGURE 3:**
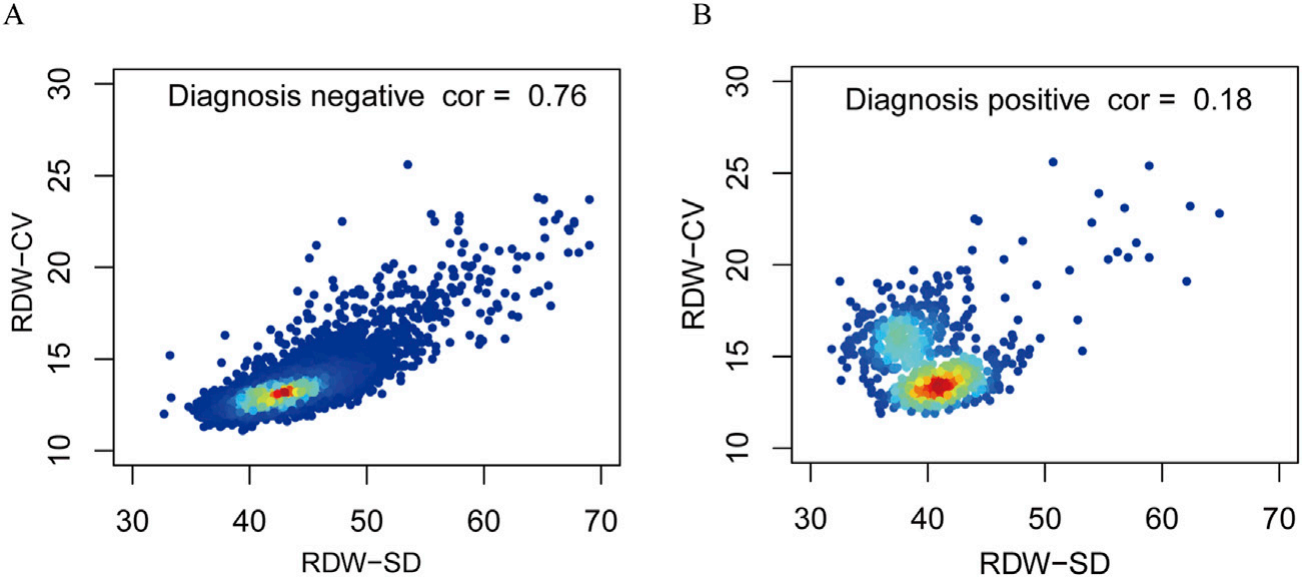
Correlation between RDW-CV and RDW-SD of the negative diagnosis populations and positive diagnosis populations. Correlations of negative diagnosis populations **(A)**. Correlations of positive diagnosis populations **(B)**.

Figure 3 shows the relation between DW-CV values and RDW-SD values among the subjects examined. The DW-CV levels increased significantly with RDW-SD values in negative populations, which aligns with the direct proportional relationship between the two variables. However, no significant correlation was found between DW-CV values and RDW-SD values in positive populations, suggesting an uneven size of red blood cells in patients with thalassemia.

### 3.6 Multivariable models for the diagnosis of thalassemia

Multivariable logistic regression analysis was conducted to show the impact of hematological parameters on the diagnosis of thalassemia (Table 4). The results showed that abnormal values RDW-CV did not have a statistically significant impact on the detection rates. Interestingly, individuals with abnormal MCV and MCH values had the highest genetic diagnosis rate of anemia, with the odds ratio of 36.9 (95%CI: 20.60–66.10, *p* < 0.001) and 10.22 (95%CI: 4.07–25.64, *p* < 0.001) respectively. Additionally, we observed that subjects with abnormal Ferritin levels had a lower risk of positive genetic diagnosis compared to those with normal levels, with the odds ratio of 0.04 (95%CI: 0.02–0.10, *p* < 0.001).

**TABLE 4:** Odds radio of age and hematological parameters in thalassemia.

Variables	Beta	S.E	Z	*p*-value	ORCI
Hb	0.4	0.16	2.53	0.01	1.49 (1.09–2.03)
MCV	3.61	0.3	12.13	<0.001	36.90 (20.60–66.10)
MCH	2.32	0.47	4.95	<0.001	10.22 (4.07–25.64)
RDW.SD	0.94	0.15	6.28	<0.001	2.56 (1.91–3.42)
RDW.CV	0.07	0.32	0.21	0.837	1.07 (0.57–2.01)
Ferritin	−3.15	0.46	−6.9	<0.001	0.04 (0.02–0.10)

## 4 Discussion

Thalassemia is a common monogenetic genetic disease that can impose significant psychological pressure on expectant couples (Taher et al., 2018; Harteveld et al., 2022). Early identification and diagnosis of thalassemia, especially during pregnancy, are crucial foreffective clinical management and guiding fertility decisions based on genotype-phenotype correlations through genetic counseling. In this study, we report the molecular characteristics and phenotypic features of thalassaemias in pregnant women. Our results show that the thalassemia types of pregnant women in Changsha County are α-thalassemia (72.19%, 1,594/2,208), β-thalassemia (26.18%, 578/2,208) and combined α + β (1.63%, 36/2,208)), the rank of prevalence rates are α-thalassaemia (4.107%), β-thalassaemia (1.489%) and α + β-thalassaemia (0.093%). In addition, our study also corresponds to the genotype and phenotype relationship between thalassemia mutations in genetically confirmed pregnant women, which is of great significance for providing guidance for prenatal diagnosis and genetic counseling for pregnant women (Ahmed et al., 2002; Shang et al., 2017).

The data from this study align with previous research findings, indicating that α-thalassemia was the predominant diagnostic type in the population (Yu et al., 2022). The proportion of α-thalassemia mutations was nearly four times higher than that of β-thalassemia mutations. However, it is noteworthy that the prevalence of positive α-thalassemia diagnosis in our population (4.107%) was significantly lower compared to previously reported rates in the Yunnan region (13.93%), Guangdong region (8.53%), Jiangxi region (7.88%), and Hunan region (5.97%) (Luo et al., 2022; Huang et al., 2020; Xu et al., 2004; Yang et al., 2023). One possible reason for the decline in the overall diagnosis rate could be that the screening program targets all pregnant women including some carriers with no counselling needs or mild symptoms, which in turn reduces the proportion of positive diagnosis rates. As a result, our study offers a more precise depiction of the overall prevalence and genotype distribution among the pregnant population in our locality. The limitation in generalizing our findings to other regions lies in the scope of our screening program, which may not be feasible in areas with larger populations. Future research should employ appropriate sampling methods to establish representative cohorts and assess the true prevalence of thalassemia in different regions and populations.

In our study, we identified that α-thalassemia is mainly caused by three types of gene deletions, including -α^3.7^/αα, -@-^SEA^/αα and -α^4.2^/αα, which was different from that of Hunan with fewer -α^3.7^/αα and more -@-^SEA^/αα (He et al., 2017b). Besides these common mutation types, we also found some rare mutations. CD 30 -GAG [-Glu]/αα ntsGAG, a rare variant caused by the deleted from codon 30 of the α2-globin gene. This variant has the potential to cause hydrops fetalis in fetuses and prenatal diagnosis and termination of such affected pregnancies are advisable (Chan et al., 1997; Chan et al., 1988). And @-THAI was reported in southern China was also detected in this study (He S. et al., 2017). Carriers of this variant may exhibit mild α-thalassemia symptoms similar to those of α^0^ thalassemia. If hemoglobin levels are low, both partners should undergo further genetic testing for thalassemia, and prenatal testing for the fetus should also be considered if necessary (Farashi and Harteveld, 2018). In addition, other rare mutations had also been identified, such as CD 61 AAG > TAG [Lys > STOP]/αα, α^fusion^/αα, Init CD (-T)/αα, Hb Amsterdam-A1/αα, and CD 108 ACC > AAC [Thr > Asn]/αα. Pregnant women carrying these rare mutations should have their partners undergo genetic testing to determine if they are at high risk for thalassemia. Couples identified as high risk are advised to consider prenatal diagnosis (Xi et al., 2024). The rare mutations further confirm the necessity for prenatal diagnosis of thalassemia in pregnant women.

β-Thalassemia are mainly caused by point mutations, which exhibiting great heterogeneity in molecular defects and clinical phenotypes. In China, more than 145 different β-thalassemia gene mutations have been detected (Yang et al., 2023), the most two frequent β-thalassemia mutations are CD 41/42 (-TTCT)/β^N^ and IVS-II-654 (C>T)/β^N^ that account for 65.31% of the genotypes (Luo et al., 2022). Our result showed that IVS-II-654 (C>T)/β^N^ and 41/42 (-TCTT) were the most common mutations, which was analogous to the research published in the Hubei provinces, Guangdong provinces, and Jiangxi provinces (Zhu et al., 2020; Yin et al., 2014; Lin et al., 2014). The ranking order of the two major mutations were also agreed with previous study in Hunan provinces, which is most likely due to the close relationship between these populations in Hunan provinces and neighboring provinces (Luo et al., 2022). In addition to common β-thalassemia mutants, we also detected some rare β-thalassemia deletion variants, such as 5′UTR +43 to +40 (−AAAC)/β^N^, −50 (G>A)/β^N^, and SEA-HPFH/β^N^, which accounted for 10.035% of all β-thalassemia mutations. The 5′UTR +43 to +40 (−AAAC)/βN mutation is a transcriptional variant that leads to a mild to minimal reduction in β-globin levels and is occasionally considered ‘silent’ (Thein, 2018). Carriers of this mutation generally do not require specific treatment, and prenatal diagnosis for the fetus is usually not necessary. Additionally, for pregnant women diagnosed with rare β-thalassemia mutations, prenatal diagnosis should be offered if they have previously had a child with severe thalassemia. Following confirmation of the diagnosis, both partners should receive genetic counseling, and selective pregnancy termination may be considered with informed consent (Liao et al., 2005).

In this study, we identified a total of 8 rare α-thalassemia genotypes and 11 rare β-thalassemia genotypes. Notably, patients with -@-^THAI^/αα and SEA-HPFH/β^N^ genotypes showed a significant decrease in average MCV and MCH values. Hence, it is recommended to include rare mutations that lead to substantial changes in blood routine indexes in traditional genetic testing. Interesting, all α^0^‐thalassemia carriers and β^0^‐thalassemia carriers had been documented with abnormal red blood cell indices in our study. Moreover, the sizes of red blood cells were found to be uneven in patients with a positive genetic diagnosis, which indicates that the genetic diagnosis results and hematology diagnosis results maintained good consistency. However, we observed no changes in hematological parameters in a subset of individuals who tested α^+^‐thalassemia carriers and β^+^‐thalassemia carriers. This suggests the necessity of considering more comprehensive and effective molecular testing methods to identify pregnancies at risk of having fetuses with thalassemia disease. Unexpectedly, during our analysis of the relationship between genetic diagnosis rate and blood abnormal parameters using multiple regression, we observed a higher positive diagnosis rate in pregnant women who exhibited multiple abnormal hematological parameters, specifically MCV and MCH. These findings suggest that screening for thalassemia using combined MCV and MCH tests can effectively enhance the diagnostic rate of genetic testing. In fact, we observed that approximately 65% of pregnant women carrying alpha- and beta-thalassemia mutations had abnormal hematological parameters or Hb electrophoresis results. Therefore, from an antenatal care perspective, it is essential to conduct genetic screening for pregnant women with abnormal blood counts to rule out the possibility of thalassemia. Additionally, we found that approximately 2.2% of individuals with normal hematological parameters and/or Hb electrophoresis results were identified as carriers of alpha- and beta-thalassemia mutations through NGS testing. This finding underscores the risk of missed diagnoses when relying solely on routine blood tests, highlighting the necessity for genetic screening to improve the detection rate of thalassemia. Interestingly, we also found that pregnant women with abnormal Ferritin levels had a lower rate of positive genetic diagnosis. The findings imply that iron deficiency anemia could potentially lead to misdiagnosis in genetic testing results.

Our study employed next-generation sequencing (NGS) for the detection of thalassemia. Despite its higher cost and the complexity of bioinformatics analysis, NGS offers broader detection capabilities compared to traditional methods like Gap-PCR and mutation-specific PCR, thereby reducing the risk of missed diagnoses. Recent advancements in NGS technology have significantly lowered the cost to approximately $10 per sample (Yang et al., 2023), enhancing its value in prenatal screening. Although third-generation sequencing (TGS) methods for thalassemia detection are emerging, their high cost and time-consuming nature currently limit clinical adoption. Thus, socioeconomic factors influence the choice of screening methods, and comprehensive healthcare policies are essential to improve the detection rate of thalassemia.

This study identified a diverse range of rare thalassemia mutations, which is in line with the location and the growing population in Changsha county. It is worth noting that prenatal screening using Next-generation sequencing (NGS) technology offers greater sensitivity compared to traditional detection methods (He et al., 2017b; Lohmann and Klein, 2014). As a result, our detection process revealed many more rare thalassemia mutations than common thalassemia mutations. Despite the cost and time involved in NGS, it can enhance the accuracy of diagnosis.

## 5 Conclusion

This study is the first to apply NGS to comprehensively profile the genotypes of thalassemia in pregnant women in Changsha county. The findings contribute to a better understanding of the thalassemia mutation spectrum in Hunan province and offer valuable insights for genetic counseling and prenatal diagnosis of thalassemia among local pregnant women. Additionally, phenotypic features of thalassemia were analyzed, providing further insights into the spectrum of genotypes in pregnant women. Furthermore, the study explored the relationship between genotype and phenotype, revealing that certain abnormal blood parameters have a remarkably impact on genetic diagnosis results. This suggests that the diagnosis approach, which combines hemoglobin electrophoresis and NGS, is beneficial in reducing the misdiagnosis rate.

## Data Availability

The data analyzed in this study is subject to the following licenses/restrictions: The original contributions presented in the study are included in the article, further inquiries can be directed to the corresponding authors. Requests to access these datasets should be directed to MZ, 11034236@qq.com.
